# Involvement of caspase-3 in stilbene derivatives induced apoptosis of human neutrophils *in vitro*

**DOI:** 10.2478/v10102-012-0013-6

**Published:** 2012-06

**Authors:** Tomáš Perečko, Katarína Drábiková, Radomír Nosáľ, Juraj Harmatha, Viera Jančinová

**Affiliations:** 1 Institute of Experimental Pharmacology & Toxicology, Slovak Academy of Sciences, SK-84104 Bratislava, Slovakia; 2 Institute of Organic Chemistry and Biochemistry, Academy of Sciences of the Czech Republic, v.v.i., Flemingovo namesti 2, 166 10, Praha, Czech Republic

**Keywords:** apoptosis, caspase-3, neutrophils, stilbene derivatives

## Abstract

Chronic inflammatory diseases, e.g. rheumatoid arthritis or cystic fibrosis, are characterised by neutrophil infiltration in inflamed tissues. Dysregulated neutrophil death may contribute to the pathogenesis of diseases where neutrophils play a role. Stilbene derivatives are reported to activate apoptosis in different cell lines. Neutrophils from healthy volunteers were incubated in vitro with resveratrol, pterostilbene, pinosylvin or piceatannol (1–100 µmol/l), and cytotoxicity and apoptosis were measured by luminometry and flow cytometry, respectively. Enhancement and/or inhibition of human recombinant caspase-3 enzyme activity were measured by luminometry. None of the stilbene derivatives tested increased ATP liberation from human neutrophils, thus showing no direct cytotoxicity effect. Resveratrol and piceatannol (100 µmol/l) treated neutrophils had a higher rate of apoptosis compared to non-treated cells. Pterostilbene and pinosylvin (1 µmol/l), yet not resveratrol or piceatannol, increased the activity of caspase-3. However in the concentration of 100 µmol/l, all stilbene derivatives tested inhibited caspase-3 activity. Their effects on human neutrophil apoptosis differed according to the structure of the molecule. Additional studies are required to get insight into the mechanisms involved in the effects of the substances tested on neutrophil viability.

## Introduction

Neutrophils are part of the innate immune system. After activation they adhere to and migrate through the endothelium. In response to infection or tissue injury, neutrophils produce reactive oxygen species to destroy invading microorganisms or foreign particles. On the other hand, activated neutrophils contribute to tissue damage by producing oxygen intermediates in chronic inflammatory diseases (Segel *et al.*, 2011). Thus the processes controlling the life-span of short-lived neutrophils are important, yet less well understood. Nowadays, significant research interest is focusing on apoptosis in treating neutrophil-dominant inflammatory diseases, *e.g.* rheumatoid arthritis, cystic fibrosis or ischaemia-reperfusion tissue injury (Fox *et al.*, 2010). There is evidence from animal models indicating that the promotion of neutrophil apoptosis may promote resolution of inflammation (Fox *et al.*, 2010). Programmed cell death – apoptosis – is an important process for successful removal of recruited neutrophils. Neutrophils express high levels of the pro-apoptotic proteins, *e.g.* Bax, Bak, Bid, Bad and Bim (Witko-Sarsat *et al.,* 2011). On the other hand, neutrophils do not express the anti-apoptotic protein Bcl-2 (Elbim and Estaquier, 2010) and marginally express the anti-apoptotic proteins A1, Mcl-1 and Bcl-X_L_ (Simon, 2003). After translocation of some pro-apoptotic proteins into the mitochondrial membrane and subsequent cytochrome c release, caspase-3 is activated and neutrophils are undergoing apoptotic death (Simon, 2003). Caspase-3 belongs to the effector group of caspases, which are responsible for the executive phase of apoptosis (Fan *et al.*, 2005). Caspase activation from their pro-caspase form has been widely described in cells undergoing apoptosis, including neutrophils.

There is considerable evidence of pro-apoptotic effects of different natural polyphenolic compounds on cultured cell lines or cancer cells (Oz and Ebersole, 2010; Singh *et al.*, 2011). Resveratrol and pterostilbene were found to induce the mitochondrial apoptotic signal Bax in cancer cells (Chakraborty *et al.*, 2010; Gogada *et al.*, 2011). Transfer of Bax into the outer membrane of mitochondria and subsequent activation of caspase-3 plays a role also in human neutrophil death. Pan *et al.* (2007) and Alosi *et al.* (2010) reported that pterostilbene treatment increased caspase-3 and -7 activity and apoptosis in different cancer cell lines. Piceatannol showed concentration-dependent induction of cell death in HL-60 cells due to activation of caspases -3, -8 and -9 (Chowdhury *et al.*, 2005).

**Figure 1:**
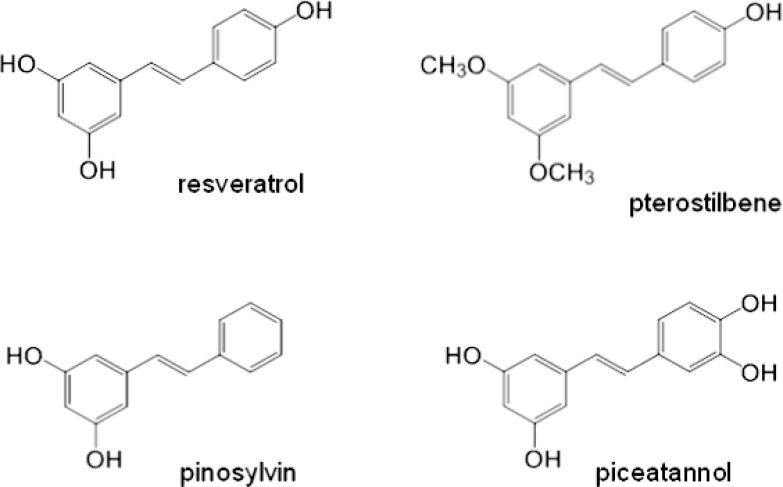
Structures of resveratrol, pterostilbene, pinosylvin and piceatannol.

In this paper we examined the effects of stilbene derivatives – resveratrol, pterostilbene, pinosylvin and piceatannol (Figure 1) on human neutrophil cell death *in vitro,* with particular interest in the involvement of caspase-3 in the apoptosis process. Along with other authors, we believe that the induction of neutrophil apoptosis is a powerful pro-resolution event which may terminate inflammation and promote tissue homeostasis (Duffin *et al.,* 2010).

## Materials and methods

Resveratrol, pinosylvin and pterostilbene were synthesised in the Institute of Organic Chemistry and Biochemistry, Prague, Czech Republic. Piceatannol, D-luciferin and luciferase were purchased from Sigma, Germany. FITC-conjugated annexin-V and propidium iodide were from eBioscience (Vienna, Austria). Caspase-Glo 3/7 Assay was from Promega (Madison, USA), human purified caspase-3 was from Enzo Life Sciences (Lausen, Switzerland). All other products are available commercially or their origin is mentioned in the text.

### Isolation of neutr ophils

Blood from healthy volunteers was collected in 2×9 ml citrate tubes. Neutrophils were isolated by 3% dextrane centrifugation and separated on Lymphoprep (Fresenius, Norway) (Jančinová *et al.*, 2006). The erythrocytes were removed with hypotonic cold haemolysis. Cells were washed with phosphate-buffered saline (PBS) before counting on Coulter Counter (Beckman Coulter). Concentration of cells in suspension was adjusted according to procedure used.

### Dose-response toxic effect of substances tested

To investigate the cytotoxic effect of resveratrol, pterostilbene, pinosylvin or piceatannol on human neutrophils we used ATP liberation test. Per sample 30 000 neutrophils were incubated with 1, 10 or 100 µmol/l concentration of substances tested for 15 minutes in dark (37 °C). Then 10 µl of luciferin-luciferase solution was added and chemiluminescence was immediately measured on Luminometer Immunotech LM-01T for 60 seconds. The amount of released ATP was calculated from the calibration curve. Sample of sonicated neutrophils (10 s, 20 kHz, Sonopuls HD 2070) was used as positive control for ATP liberation.

### Analysis of apoptosis

The apoptosis assay was evaluated using leukocyte-rich plasma fraction of human blood. One ml of buffy coat was collected and stored on ice before use. The cells were counted on haemocytometer and 200 000 neutrophils per sample were used. Three different concentrations of stilbene derivatives (1, 10 and 100 µmol/l) and a control sample were incubated at 37 °C for 10 min. The cells were stained with annexin-V conjugated with FITC in dark at 4 °C for 10 min, followed by staining with propidium iodide (1 µg/ml) and analysed immediately by Beckman Coulter Cytomics FC500 cytometer. All samples were analysed under the same conditions (gains, volts). From the granulocytic area, 5 000 cells were gated and analysed. Only annexin-V positive cells were considered pre-apoptotic cells and double positive cells (annexin-V+/propidium+) were considered late-apoptotic or dead cells (Jančinová *et al.*, 2011).

### Caspase-3 activity

Following caspase cleavage of the Z-DEVD-aminoluciferin substrate (resulting in the reaction of luciferase with amino-luciferin) the measurement of light production was made on the Luminometer Immunotech LM-01T. According to the manufacturer′s instructions, 10 µl of 0.1 IU caspase-3 (EC3.4.22.56) was added to 20 µl aliquots of different stilbene derivatives concentrations and buffer solution. Finally 50 µl of Caspase-Glo 3/7 Reagent was added, the mixture was measured for 60 minutes and the activity of caspase-3 was identified. The different concentrations of vehiculum solution were also tested.

### Statistics

Data were examined using the Student t-test and *p-*values below 0.05 were considered statistically significant. Standard Error of the Mean (SEM) was calculated as standard deviation divided by square root of number of samples.

## Results

**Figure 2:**
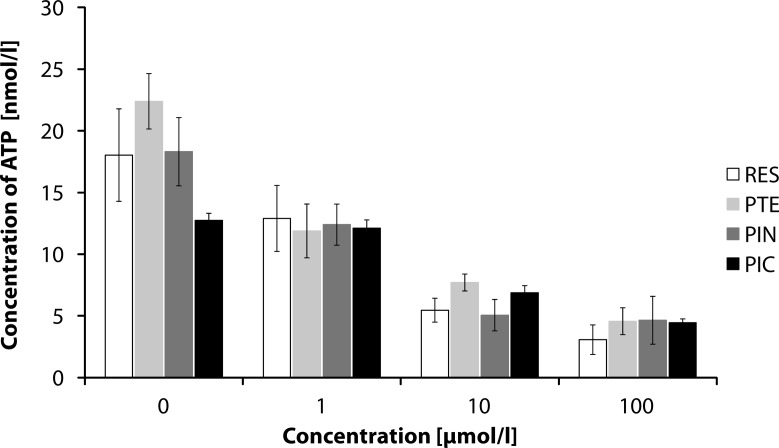
Effect of 1, 10 or 100 µmol/l of resveratrol (RES), pterostilbene (PTE), pinosylvin (PIN) or piceatannol (PIC) on ATP release from human neutrophils shown as nmol/l of ATP. 0 – control sample. Mean ± SEM, n = 6–8.

We investigated the possible cytotoxic effects of resveratrol, pterostilbene, pinosylvin and piceatannol on human neutrophils. Release of ATP from cells can be used as a marker of cytotoxicity (Crouch *et al.*, 1993). By using the luciferin-luciferase method for ATP detection, we showed no acute cytotoxic effects of the stilbene derivatives tested on human neutrophils (Figure 2). The value of liberated ATP in positive control sample (sonicated neutrophils) ranged from 200 to 400 nmol/l.

**Figure 3:**
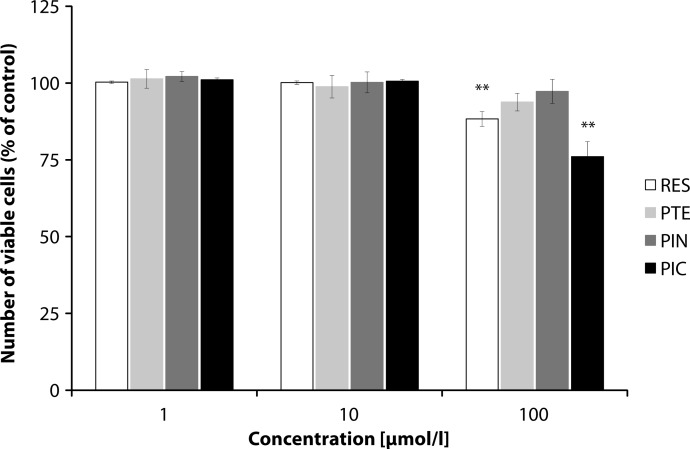
Number of viable neutrophils after 10 min / 37 °C incubation with 1, 10 or 100 µmol/l of resveratrol (RES), pterostilbene (PTE), pinosylvin (PIN) or piceatannol (PIC) shown as % of control sample (100%). Mean ± SEM, n = 5–8. **p<0.05 vs control sample.

We wanted to determine if the derivatives tested induced neutrophil death through apoptosis. In our experiment, resveratrol in 100 µmol/l concentration decreased the viability of human neutrophils to 88% (*p<*0.01) compared to non-treated cells (Figure 3). The same concentration of piceatannol was more effective than resveratrol, decreasing the number of viable neutrophils to 76% (*p<*0.01). In the lower concentrations tested, there were no effects on neutrophil apoptosis. Pterostilbene and pinosylvin did not change the number of viable neutrophils significantly in any concentration tested (Figure 3).

**Figure 4:**
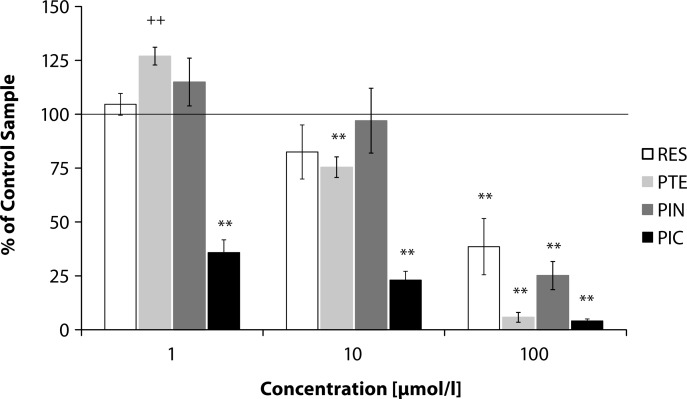
Effect of 1, 10 or 100 µmol/l of resveratrol (RES), pterostilbene (PTE), pinosylvin (PIN) or piceatannol (PIC) on caspase-3 activity shown as % of control sample (100%). Mean ± SEM, n = 4–5. ++*p<*0.05 vs control sample for activation, **p<0.05 vs control sample for inhibition.

There are many studies showing the promoting role of caspase-3 in spontaneous and mediated apoptosis in neutrophils (Pongracz *et al.*, 1999, Weinmann *et al.*, 1999). We observed that incubation of human recombinant caspase-3 with resveratrol for 60 minutes resulted in decreasing activity of this enzyme in a concentration dependent manner (Figure 4), with significant values on using resveratrol in 100 µmol/l concentration. Piceatannol inhibited the activity of human recombinant caspase-3 in 1, 10 and 100 µmol/l concentration (*p<*0.01). Pterostilbene (1 µmol/l) showed significant (*p<*0.01) increase of caspase-3 activity. However, in the concentrations of 10 and 100 µmol/l, pterostilbene inhibited (*p<*0.01) the enzyme activity. On using 1 µmol/l of pinosylvin, the increase of caspase-3 activity was not significant. On the other hand, 100 µmol/l pinosylvin induced significant (*p<*0.01) inhibition of the enzyme. The effect of pinosylvin was comparable to that of pterostilbene, yet to a lesser extent (Figure 4).

## Discussion

Although there is considerable evidence of pro-apoptotic effects of some stilbene derivatives on cultured cell lines or cancer cells (Ko *et al.*, 2011; Liu *et al.*, 2011; Radhakrishnan *et al.*, 2011; Zhou *et al.,* 2011), little is known about the effects of these compounds on human neutrophil viability and apoptosis. Resveratrol is a well known stilbene derivative with pro-apoptotic effects. We investigated the effects of resveratrol and its congeners – pterostilbene, pinosylvin and piceatannol on human neutrophil viability *in vitro*. Neither of the compounds tested was cytotoxic, as verified by the ATP cytotoxicity test. From the four derivatives of stilbene tested, only resveratrol and piceatannol in the highest concentration used (100 µmol/l) decreased significantly the viability of human neutrophils. Addition of another hydroxyl group into the molecule structure of resveratrol led to higher pro-apoptotic effect of piceatannol, resulting in 24% of apoptotic neutrophils compared to 12% in resveratrol treated neutrophils. This is in agreement with other studies suggesting that the number and position of hydroxyl groups play a role in pro-apoptotic effects of stilbene derivatives (Horvath *et al.,* 2007). Interestingly, only annexin-V positive neutrophils were present with no increment in propidium iodide positive cells (data not shown). Annexin-V detects phosphatidylserine molecules in the outer membrane of apoptotic cells whereas propidium iodide binds to DNA. Propidium iodide is not membrane-permeable and generally excluded from viable cells. It is widely used for identifying dead cells in a population (Ormerod, 2005). Considering also the results from ATP cytotoxicity test, we may conclude that resveratrol and piceatannol promoted apoptosis of neutrophils rather than killing the cells. Next we investigated the mechanism of stilbene-induced apoptosis in human neutrophils on caspase-3 level. Caspase-3 belongs to the effector group of caspases which are responsible for the executive phase of apoptosis (Fan *et al.*, 2005). Caspase activation from their pro-caspase form has been widely described in cells undergoing apoptosis, including neutrophils. Avdi *et al.* (2001) observed a time-dependent cleavage of pro-caspase-3 to a caspase-3 cleavage product in lysates from adherent neutrophils that had been exposed to TNFa. In our experiment we focused on the activity of the active form of human recombinant caspase-3 enzyme. It is obvious that the number and position of hydroxyl or methoxyl groups in the structure of stilbene derivatives are playing a role in caspase-3 activation and/or inhibition. Though pterostilbene and pinosylvin failed to affect neutrophil apoptosis, only these two derivatives increased caspase-3 activity in 1 µmol/l concentration. With higher concentrations tested, this effect diminished, showing inhibition of caspase-3 activity. In contrast to our results on purified caspas-3 enzyme, Pan *et al.* (2007) and Alosi *et al.* (2010) reported that pterostilbene treatment increased caspase-3 and -7 activity and apoptosis in different cancer cell lines in a concentration- and time-dependent manner. In our experiments with caspase-3, resveratrol and piceatannol inhibited the activity of this enzyme, which is in contrast with their pro-apoptotic effects examined in human neutrophils. However, in HL-60 cell line, widely used in studies replacing human neutrophils, piceatannol showed concentration-dependent induction of cell death due to activation of caspases-3, -8 and -9 (Chowdhury *et al.*, 2005). On the one hand, stilbenes were found to exert higher apoptosis-inducing activity against tumour cell lines than against normal cells (Chowdhury *et al.,* 2005). On the other hand, there could be differences in the effects of stilbene derivatives on the activity of purified caspase-3 enzyme compared to the activity of this enzyme within the cell. White *et al.* (2011) have suggested two explanations to account for the different effects: the polyphenol tested was being degraded in the cytoplasm of the cell or was not even able to effectively cross the cell membrane and enter the cell. But how do resveratrol and piceatannol induce apoptosis in the context of their ability to inhibit caspase-3? Our results suggest a caspase-3-independent form of cell-death (Kroemer and Martin, 2005). Finally, several data indicate also an important role of redox signalling in neutrophil apoptosis (Kasahara *et al.*, 1997; Hampton *et al.*, 2002). In human neutrophils, we reported scavenging of reactive oxygen species on using stilbene derivatives (Perecko *et al.*, 2008).

Our aim is to contribute to the understanding of processes influencing the life span of human neutrophils that may result in the development of novel therapeutics for inflammatory diseases. Along with other authors, we believe that the induction of neutrophil apoptosis is a promising pro-resolving effect that may have potential benefits for treating chronic inflammatory diseases.
